# A high-resolution database of historical and future climate for Africa developed with deep neural networks

**DOI:** 10.1038/s41597-025-05575-8

**Published:** 2025-07-22

**Authors:** Sarah A. Namiiro, Andreas Hamann, Tongli Wang, Dante Castellanos-Acuña, Colin R. Mahony

**Affiliations:** 1 Department of Renewable Resources, University of Alberta, 751 General Services Building, Edmonton, AB T6G 2H1 Canada; 2 Centre for Forest Conservation Genetics, Department of Forest and Conservation Sciences, University of British Columbia, Vancouver, BC, Canada; 3 British Columbia Ministry of Forests, Victoria, BC Canada

**Keywords:** Climate and Earth system modelling, Projection and prediction

## Abstract

This study contributes an accessible, comprehensive database of interpolated climate data for Africa that includes monthly, annual, decadal, and 30-year normal climate data for the last 120 years (1901 to present) as well as multi-model CMIP6 climate change projections for the 21^st^ century. The database includes variables relevant for ecological research and infrastructure planning, and it comprises more than 25,000 climate grids that can be queried with a provided *ClimateAF* software package. In addition, 30 arcsecond (~1 km) resolution gridded data are available for download. The climate grids were developed with a three-step approach, using thin-plate spline interpolations of weather station data as a first approximation. Subsequently, a novel deep learning approach is used to model orographic precipitation, rain shadows, lake and coastal effects at moderate resolution. Lastly, lapse-rate based downscaling is applied to generate high-resolution grids. The climate estimates were optimized and cross-validated with a checkerboard approach to ensure that training data was spatially distanced from validation data. We conclude with a discussion of applications and limitations of this database.

## Background & Summary

With increasing concern over climate change, interpolated climate data have become essential for assessing climate change impacts, monitoring natural and managed ecosystems, and designing conservation and mitigation strategies to minimize climate change impacts. Virtually every study in the field of climate change impacts and adaptation requires information on long-term climate conditions (30-year climate normals), records of past climate variability (monthly, seasonal, and annual historical data), as well as future projections from Atmosphere-Ocean General Circulation Models (AOGCMs). Such data are now widely available at global scales, but they also vary widely in quality among continents and regions depending on weather station coverage, as well as in the methodological approaches that were used to generate interpolated climate products.

Gridded climate products are generated by broadly three methodologies. The first approach is direct interpolation of ground-based weather station data using techniques such as kriging, inverse distance weighting and splines. Examples include widely used products such as WorldClim^1,2^, and the Climatic Research Unit gridded Time Series (CRU-TS) grids^3,4^. The second approach uses AOGCMs run on historical weather station data, the same models that provide daily and hourly weather forecasts. Gridded historical climate data generated with this approach is referred to as a reanalysis product. Examples of widely used global reanalysis products are ERA5 (European Centre for Medium-Range Weather Forecasts Reanalysis v5)^5^, and its derivative CHELSA (Climatologies at High resolution for the Earth’s Land Surface Areas)^6^, which downscales ERA5 data to higher resolutions. A third approach uses satellite-based remote sensing, calibrated with ground-based weather station data, to estimate precipitation and temperature variables. Examples include the global Climate Hazards Group InfraRed Precipitation with Station data (CHIRPS)^7,8^ and the temperature counterpart, CHIRTS^9^.

While such global products are widely used, regional efforts that take advantage of additional station data, serve specific local needs, or that are optimized to capture particular regional climate patterns are also well-regarded. For example, the Parameter-elevation Relationships on Independent Slopes Model (PRISM) for the United States utilizes topographic information to model small-scale weather patterns such as temperature inversions in mountain valleys, precipitation induced by orographic lift and rain shadows^10^. For Africa, regional comprehensive climate databases are lacking, although some products exist for specialized applications, including the Africa Rainfall Climatology for famine early warning systems (ARC2)^11^, the University of Reading’s TAMSAT African Rainfall Climatology And Time series (TARCAT)^12^. Both ARC2 and TARCAT combine satellite imagery and interpolation of climate station data to obtain high-quality precipitation grids for regions with relatively low weather station density.

Both global and regional products make compromises in the extent of their spatial and temporal resolution, climate variable coverage, inclusion of future projections, and coverage of historical time periods for computational reasons and data management limitations. To address these limitations, Wang, *et al*.^13^ and Marchi, *et al*.^14^ developed software packages for North America and Europe that build on a 2.5 arcminute medium-resolution 1961–1990 climate baseline, developed with the Parameter-elevation Relationships on Independent Slopes Model (PRISM) by Daly *et al*.^10^. The software subsequently uses local environmental lapse rates to dynamically downscale the climate data to any user-selected resolution based on a digital elevation model (with the finest recommended resolution being 250 m in mountainous terrain). Alternatively, the software can provide point estimates of climate variables based on user-provided point coordinates. If the coordinates also have an elevation recorded, the software will provide a scale-free climate estimate using local environmental lapse rate adjustment.

In prior work as well as this study, the 1961–1990 climate normal period was chosen as a reference period for the 2.5 arcminute medium-resolution baseline because this period has the highest number of weather station records^14,15^. However, the methodology is not sensitive to the choice of any specific baseline, and any other historical normal period (from 1901 to the present) can be generated using the delta (change factor) method by overlaying lower resolution anomaly layers (0.5 degree), thereby minimizing data storage requirements, while maintaining the statistical accuracy of a high-resolution database. Similarly, future climate projections can be generated by the software, using the same change-factor approach, which in this case also serves as an effective bias correction method for AOGCM estimates^13,16^.

PRISM baseline data or comparable products are not available outside of North America and Europe. In order to develop an equivalent software package for Africa that is capable of modeling local weather patterns in complex topographic terrain, we employ deep neural network methodology that has been shown to be a promising approach to capturing complex local climate patterns that are non-linear and the result of multiple interacting topographic and atmospheric factors^17,18^. The novelty of our approach lies in integrating deep learning in a stepwise interpolation and downscaling process, where different methods are employed for tasks that reflect their strengths. As a first step, thin-plate spline interpolation of weather station data provides an efficient first approximation of climate estimates. Secondly, the initial estimates are fine tuned with deep learning that incorporates additional geographic information (such as elevation, aspect, slope, distance to coast and lakes) in combination with atmospheric data from a general circulation model (monthly wind direction and strength) to model local weather patterns at moderate resolution (2.5 arcminutes). The neural network estimates for the 1961–1990 baseline climate grids are then combined with low resolution historical or future anomaly grids to make adjustments for different time periods of interests (months, years, decades or normal periods). As the last step, we employ lapse-rate based dynamic downscaling with a software solution to arrive at gridded data at user-defined resolutions or scale-free point estimates of climate variables.

Here, we expand the availability of our climate databases to Africa, providing a variety of standard and bioclimatic variables for the historical time periods from 1901 to the present, and including monthly, seasonal, annual, decadal and 30-year climate estimates as well as future projections from 13 AOGCMs selected from the CMIP6 collection for various quality criteria according to Mahony, *et al*.^16^. To further aid users in selecting a representative set of future projections for different regions in Africa, we employ the Katsavounidis-Kuo-Zhang (KKZ) algorithm to maximize the representation of uncertainty in future projections by different AOGCMs for different regions of Africa.

## Methods

### Weather station data

Weather station data were compiled for the study area between latitude 37°N and 35°S and longitude 17°W and 51°E covering eleven IPCC’s climate reference regions^19^ of the African continent. For precipitation weather stations, we rely on a global database generated by Castellanos-Acuna and Hamann^15^. For temperature weather station records for Africa we carried out a similar data compilation, data cleaning and quality control effort as described in detail by Castellanos-Acuna and Hamann^15^. Briefly, data from seven public databases^3,20–25^ were merged (Table 1), and duplicates were removed using the enhanced master station history report (EMSHR) and its vector layer^26,27^. Recorded station elevations were then compared to a digital elevation model (DEM) to identify unit errors (feet vs. meters) as well as potential location errors. Generally, stations with large elevation errors in mountainous regions were retained, but stations with large elevation errors in areas of flat topography were removed due to probable location errors. Missing and zero elevation values were replaced by the DEM value.

**Table 1:** Databases included in this study detailing their temporal extent, temporal resolution and the number of stations obtained for Africa.

Database	Temporal extent	Temporal resolution	Number of stations	Reference
Climate Research Unit Time Series (CRUTS), Version 3	1849–2023	Monthly time series	1522 (572)	Harris, *et al*.^3^
Global Historic Climate Network Monthly (GHCN-M), Version 3	1878–2017	Monthly time series	864 (176)	Lawrimore, *et al*.^20^
Global Historic Climate Network Daily (GHCN-D), Version 3	1900–2021	Daily time series	878 (344)	Menne, *et al*.^21^
World-wide Agroclimatic Data of FAO (FAOCLIM), Version 2	1902–1998	Monthly time series	846 (96)	FAO^22^
World Meteorological Organization (WMO)	1961–1992	Monthly time series	431 (256)	WMO^23^
European Climate Assessment Dataset (ECA)	1892–2018	Daily & Monthly time series	223 (13)	Tank, *et al*.^24^
National Oceanic & Atmospheric Administration (NOAA)	1949–2015	Monthly time series	131	NOAA^25^
Global monthly weather station for precipitation	1901–2010	Monthly time series	4510	Castellanos-Acuna and Hamann^15^

The number of stations in parentheses is the final number of stations that were retained after applying quality control criteria, duplicate station removal and local redundancy control.

Next, stations were ranked based on the length of the station records and their overlap with the 1961–1990 normal period, for which our baseline interpolations were carried out. Tier-1 stations had at least 27 years of records (90% of a normal period) for the 1961–1990 period, and tier-2 stations had at least 27 years of records for the 1951–1990 period. Tier-3 stations also had at least 27 years records for the 1951–2000 period. Finally, tier-4 stations had at least 15 years of records at any time from 1901 to 2020. The proportion of tiers in the final database was 35%, 9%, 11%, 45% for tiers 1 through 4, respectively. Missing values for the 1961–1990 period were then calculated using the anomaly (change factor) approach relative to CRU-TS time series data and adjusted accordingly as described in Castellanos-Acuna and Hamann^15^. If station records reported only average temperature, the average minimum and average maximum monthly temperatures were inferred from the interpolated diurnal range of CRU-TS time series data. Lastly the 1961–1990 normal estimate was obtained by averaging observed and estimated monthly climate values for this period.

**Fig. 1:**
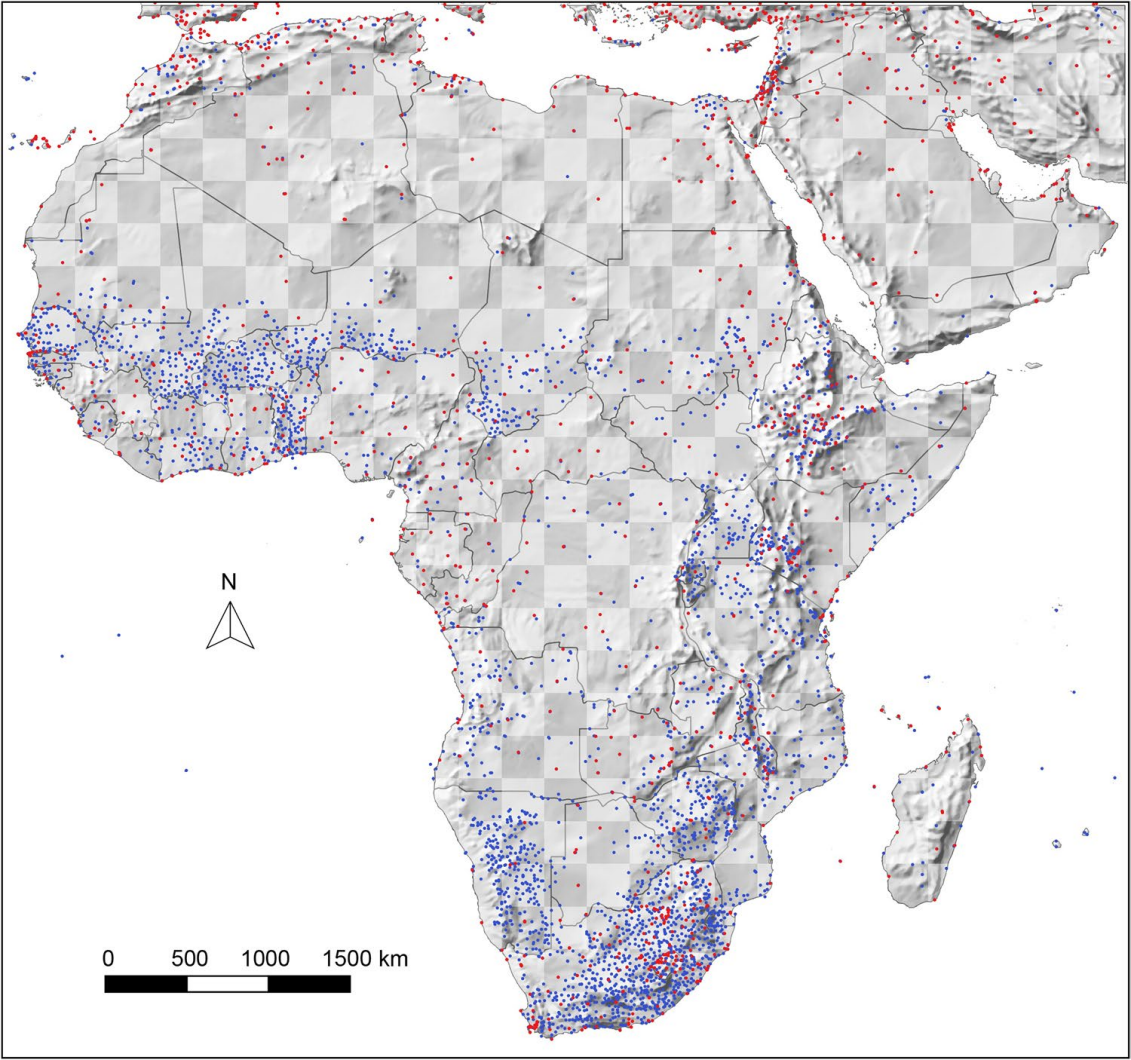
Distribution of 4625 weather stations compiled for the database. Blue stations have records for only precipitation measurements and red stations have records for both precipitation and temperature measurements for the 1961–1990 period. A three degree checkerboard pattern, used to split data for independent validation is also shown.

Lastly, we filtered stations by location, only retaining the highest-tier station per rounded 0.1 decimal degree (approximately 10 km grid cell size) and within the same 250 m elevation interval were retained. This ensured removal of lower quality station data where better records were available in close vicinity. Following this quality control process, we retained 1588 stations with temperature records and 4510 stations with precipitation records (Fig. 1). The discrepancy in counts between temperature and precipitation records is common, and arises from agricultural and meteorological programs historically prioritizing collection of precipitation data over temperature data for managing water resources, understanding irrigation needs, assessing flood risks, etc.

### Thin-plate spline interpolation

A first approximation of monthly climate grids was generated with thin-plate spline interpolation, performed using the *fastTps*() function of the *fields* package^28^ for the R programming environment (R). As a first approximation of general, location and elevation-dependent climate patterns, the interpolation was carried out on weather station data binned by three degree latitude and longitude grid cells and 250 m elevation classes (i.e. a 3D grid), with the predictor variables being the average latitude, longitude, and elevation of weather stations per 3D grid cell. This coarse 3D grid was chosen to initially only model general spatial patterns that were minimally influenced by outliers or erroneous station records. For computational efficiency, the *aRange* parameter, which controls the range of influence for the thin-plate splines, was set to 2,000 km. The R code for the TPS model used is provided in the Figshare repository^29^.

**Fig. 2:**
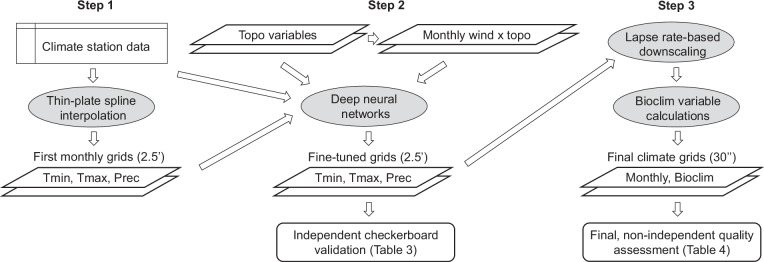
Visual summary of the novel 3-step methodological approach to climate interpolation. Thin-plate splines allow for a first approximation of monthly climate variables (Step 1). Subsequently, deep learning approach model orographic precipitation, rain shadows, lake and coastal effects (Step 2). Lastly, lapse-rate based downscaling is applied to generate high-resolution grids of monthly, seasonal, and bioclimatic variables.

Although the thin-plate spline method would in principle allow more predictor variables to be specified in the interpolation model, this approach is computationally demanding, and it would also need to be carried out on original weather station locations to capture interactions among multiple factors, simultaneously raising model complexity and the size of the dataset to be fitted. Instead we use deep neural networks, which have practically no limitations regarding model complexity and database size, to fine tune the initial interpolated climate grids with the help of additional predictor variables reflecting topographic and geographic information, such as aspect, slope, distance to coast and lakes, in combination with monthly wind direction and strength obtained from general circulation models (Fig. 2).

### Predictor variables (features)

Predictor variables, also referred to as features by the machine learning community, were generated with *Spatial Analyst Tools* in ArcGIS^30^ from a 2.5 arcminute resolution DEM (the target resolution of climate grids), which was derived from GTOPO30 data^31,32^. As potentially useful covariates besides latitude, longitude and elevation, we calculated a north-south directional hill shade, a topographic position index (TPI), which is a numerical index that describes ridges (high values), valleys (low values) and flat areas (intermediate values). A compound topographic index (CTI) is similar to TPI, but identifies valleys and ridges with a hydrological approach, where areas of convergence receive high values. The predictor variables Elevation, CTI and TPI were first transformed to be approximately normally distributed (log transformation with an appropriate constant), while the north-south directional hill shade was subjected to a bi-directional log transformation, separately for negative north-facing values and positive south-facing values to mitigate long-tailed variable distributions. Subsequently all variable values were re-scaled from 0 to 1.

Further, topographic variables weighted by wind direction were generated in a two-step process, that calculated directional exposure of geographic features, which were then scaled by average monthly wind direction and strength for the 1961–1990 period obtained from the Modern-Era Retrospective analysis for Research and Applications, Version 2 (MERRA-2)^33^.Westward and southward exposure of mountains was approximated with hill shades (45° angle with a 180° or 270° azimuth). Directional lake and coastal influences were generated with an equivalent “topography”, derived from a 2.5 arcminute grid representing lakes or oceans with a value of 1, versus land represented by a value of 0. The grid was repeatedly subjected to a 3 × 3 low pass filter to the desired range of putative lake or ocean effects (approximately 10, 50, 100 and 500 km). Directional information of distance to lake shores and coast lines were generated with hill shades as described for mountains above (180° & 270° azimuth). Both topographic and distance to waterbody “hill shades” were subsequently scaled from + 1 (e.g., maximum westward mountain exposure, or minimum westward distance to lake or coastline) to 0 (flat topography or beyond maximum distance to waterbody), to –1 (equivalent in opposite direction) and multiplied with MERRA-2 monthly wind direction and strength, provided respectively in north-south and east-west direction. To generate a single exposure layer from two directional layers, we used the geometric mean of east-west and north-south directional effects (geometric mean to avoid an overestimate of where north-south and east-west exposures overlap). The resulting grids (12 exposure layers representing each month of the year) were then again re-scaled from 0 to 1 for machine learning.

We note that deep neural networks are generally sensitive to transformation and scaling of training data, with even distributions and scaling as described above essential for good neural network performance for multiple reasons. Predictor variables need to scale with the initial random weights at a ratio of around 10:1 to achieve initial convergence and gradient decent; consistent variable scaling also prevents saturation of activation functions; and uniform initial weighting of predictors provide an unbiased starting point for training. That said, these are only consideration to improve computational performance and optimal convergence. Neural networks have no assumptions with regards to the shape of relationships of predictor and response variables or their interactions.

**Fig. 3:**
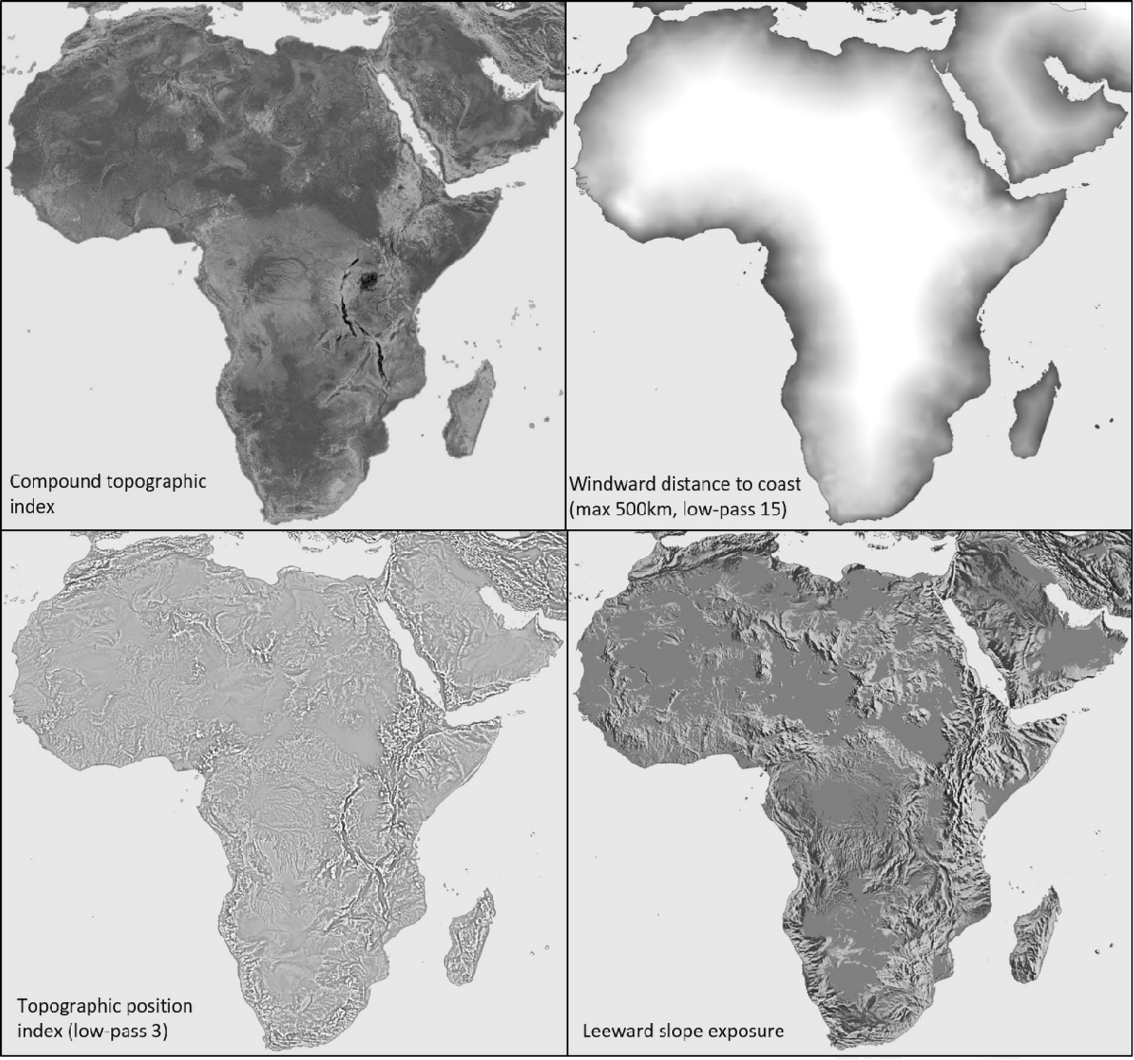
Example of predictor variables used in neural network fine-tuning of thin-plate spline interpolations. All putative predictor variables were subjected to transformations for normality if possible, and then scaled to values between 0 (black) and 1 (white) for use as covariates in deep neural network models.

As an additional modification, different versions of predictor variables were generated by subjecting them to low-pass filters of 3 × 3, 5 × 5, 7 × 7, 9 × 9 and 15 × 15 grid cells of the 2.5 arcminute target resolution. The rationale for these predictor variable versions is that topography and atmospheric circulations interact at different scales. For example, rain induced by orographic lift in mountainous regions takes place at the height of cloud layers and therefore does not closely track minor topographic variation at the ground level. Because the optimal scale is unknown, we generated a range of scales for predictor variables that were evaluated through neural network importance values (based on their empirical usage in neural network weights). Although in our study, neural network predictions were not sensitive to the exact selection of variables, variable importance values nevertheless varied for different variables in different months. For data processing and programming efficiency, we used a first pass of deep neural network training on a limited set of variables to guide the selection of predictor variables that was used for development, training and prediction of all variables, listed in Table 2, and with some examples shown in Fig. 3.

**Table 2:** Predictor variables selected for training deep neural networks.

Predictor variables for machine learning	Low-pass filter versions		
Base variables			
Thin-plate spline interpolation of climate variable			
Latitude			
Longitude			
Topographic variables			
Elevation	3	7	15
Compound topographic index	5	9	
Topographic position index	3	7	
Hill shade south-north direction	7		
Monthly variables weighted by wind direction and strength			
Windward ( + ) or leeward (–) slope exposure	5	9	15
Leeward wind-weighted distance to coast (max 50 km)	5		
Leeward wind-weighted distance to coast (max 500 km)	15		
Leeward wind-weighted distance to lakes (max 10 km)	5		
Leeward wind-weighted distance to lakes (max 100 km)	15		

The original target resolution was 2.5 arcminutes, and low-pass filters were applied to better predict larger scale climate patters driven by higher altitude air circulation patterns.

### Fine tuning with deep neural networks

Deep neural network methodology was applied to fine-tune the thin-plate spine interpolation with a larger dataset of predictor variables (Table 2), including all predictor variables for the location of weather stations, and the climate records of stations as dependent variable. We used the *Keras* package for R^34^ as a front end to define the neural network architecture, and the *DALEX* package^35^ to evaluate the importance of predictor variables in the neural network model. The computational work was executed on Google’s *Tensorflow* machine learning platform on an Nvidia’s RTX A2000 graphics card in Python. Our network architecture is an approximately 3 million parameter model that can execute training and prediction of a single variable for a continent in about 15 minutes on a modern mid-level consumer graphics card. Note, that software and package compatibility is version-sensitive. As of 2024, a compatible software chain comprises Anaconda v.2022.10 with Python v.3.9.13, *Tensorflow* v.2.10.1, Nvidia’s *cuDNN* v.8.1.0 library, the *CUDAtoolkit* v.11.2 for Python. This computational back-end is compatible with R v4.4.x and *Keras* (for R) v2.13.

The neural network architecture we used was a feed forward model that was empirically optimized by varying model parameters, and observing the resulting changes in the evolution of validation statistics throughout the training period, using an initial 80:20% random training:validation split. Model parameters that were varied included the number of hidden processing layers in the neural network (1 to 10 processing layers were tested), the number neurons per layer (8, 16, 32 … 4096 neurons per hidden layer), and increasing, decreasing or fixed numbers of neurons from first to last processing layer. As counter-measures to over-parameterization, we tested the inclusion of dropout layers where a proportion of neurons are re-set to a zero-activation state in specific intervals during training, as well as kernel regularization, which imposes constraints on the maximum value of the network weights in a processing layer.

As a general network architecture that worked well for all climate variables, we arrived at a relatively large first hidden layer, given a small set of 27 predictor variables, comprising 2048 neurons but that required an L2 kernel regularization, followed by a dropout layer (rate = 20%) and seven subsequent processing layers, each half the size of the proceeding layer (1024, 512, … 16 neurons). A number of standard hyperparameter choices that generally work well for feed forward network architecture proved satisfactory for our models as well: the ReLU activation function, which introduces a non-linearity into the model, a mini batch size of 32, which represents the number of stations processed at once before network weights are updated, a learning rate controlled by the Adam optimization function, and the mean absolute error as loss function, which is less pensive to outliers than the mean squared error. The implementation of this architecture with the Keras package for R is provided in the Figshare repository^29^. The wide initial processing layer allows the model to account for a variety of different local weather patterns found throughout the continent, without requiring spatial stationary in predictor-response variable relationships.

Over-parameterization was also controlled by the number of epochs (number of passes through the entire training dataset), which was set individually for each climate variable, ranging from 75 to 500. The tendency to overparameterize was first evaluated with a random 80% training and 20% validation data split (specified as a parameter in the neural network architecture in the Keras package). However, this type of validation is not entirely independent due to spatial autocorrelations among nearby weather stations. For an additional independent validation, we used the “checkerboard” method used for the development of WorldClim^2^ with a 3 × 3° grid to assign stations to cross-validation groups, delineated by their geographical coordinates falling within either a “black” or “white” tile (Fig. 1). This ensured that testing data were spatially distanced from training data, and the maximum epoch size for network training was set to where no further improvements were observed in validation statistics.

### Climate variables and historical periods

Climate variables include 48 monthly variables: mean minimum temperature (Tmin01 to Tmin12), mean maximum temperature (Tmax), monthly average temperature (Tave) and monthly precipitation (Prec). Bioclimatic variables include mean annual temperature (MAT), seasonal 3-month totals for precipitation (e.g. PrecDJF, PrecMAM, etc.), and 3-month means for maximum temperature (TmaxDJF, etc.), minimum temperature (TminDJF, etc.) and average temperature (TaveDJF, etc.), mean warmest month temperature (MWMT), mean coldest month temperature (MCMT), temperature difference (TD = MWMT-MCMT), mean annual precipitation (MAP), growing degree-days above 5 °C (DD > 5), heating degree-days below 18 °C (DD < 18), cooling degree-days above 18 °C (DD > 18), Hargreaves’ reference evaporation (Eref), and Hargreaves’ climatic moisture deficit (CMD). The calculation or estimation of these variables is described in detail by Wang, *et al*.^13^.

Also as described in detail by Wang, *et al*.^13^, we use a change factor approach to derive high-resolution historical time series of monthly, annual, decadal, and 30-year normal climate data for the last 120 years (1901 to present). Historical anomaly layers relative to the 1961–1990 normal period were developed from the Climatic Research Unit gridded Time Series (CRU-TS) v.4.05^3^ at a 0.5° grid size, corresponding to approximately 50 km resolution. Climate variables at this relatively coarse resolution are expected to be less accurate, especially in mountainous terrain. However, by converting the CRU-TS climate estimates to anomalies and downscaled by the *ClimateAF* software package, historical data can be generated with statistical precision comparable to high-resolution climate normal layers^13^. Storing only one high-resolution baseline climate grid, and 120 annual low-resolution anomaly layers (1901–2020) reduces the total database size by two orders of magnitude, with minimal sacrifice to statistical accuracy, even in complex terrain^13^.

### Future climate projections

Similarly, future climate projections can be generated by the software, using the same change-factor approach, which in this case also serves as an effective bias correction method for AOGCM estimates^13,16^. We used thirteen CMIP6 models, based on the six criteria used by Mahony, *et al*.^16^. Briefly, we required models to have a minimum of three historical runs, low bias relative to historical runs, and availability of estimates for both mean daily minimum temperature and mean daily maximum temperature and projections based on a complete set of future emission scenarios. In model selection, we also avoided to choose more than one model from the same institution, or otherwise closely related models. The 13 models included were ACCESS-ESM15, BCC-CSM2, CanESM5, CNRM-ESM2-1, EC-Earth3, GFDL-ESM4, GISS-E2.1, INM-CM5.0, IPSL-CM6A-LR, MIROC6, MPI-ESM1.2-HR, MRI-ESM2.0, UKESM1-0-LL. We use all SSP scenarios that were consistently available for the above models, which were SSP1-2.6, SSP2-4.5, SSP3-5.8 and SSP5-8.5. For more details about these models and the selection process, see Mahony, *et al*.^16^.

The selected 13-model ensemble had an average Equilibrium Climate Sensitivity (ECS) of 3.7 °C, with a variability spanning from 1.9 °C to 5.6 °C. This aligns with the ECS values derived from the complete CMIP6 ensemble, which also stands at 3.7 °C, with a range from 1.8 °C to 5.6 °C^36^. To arrive at recommendations for model ensemble subsets for Africa and its 11 IPCC climatic subregions^19^, candidate models were further narrowed down based on four additional criteria by Mahony, *et al*.^16^, including the requirement of an equilibrium climate sensitivity between 2 and 5 °C to avoid outlier models in a smaller set, a sufficiently high model resolution, a higher number of simulation runs, and low spatial anomalies in projections (excluding BCC-CSM2-MR, IPSL-CM6A-LR INM-CM5.0 and CanESM5 for regional ensemble recommendation). However, we made an exception for UKESM1-0-LL, which was retained as one (optional) representative of a highly sensitive projection.

To obtain ordered ensemble subsets, the remaining models (8 or 9 if including UKESM1-0-LL) were subjected to the KKZ algorithm^37^ to maximize multivariate representation of variability in future projections for a user-selected number of models as described by Cannon^38^. The KKZ algorithm orders the models with the first selection closest to the ensemble centroid, the second selection being most distant from the first, and the third and all subsequent selection being most distant to previously selected models in multivariate climate space. The procedure is sensitive to selecting outlier scenarios as the second selection, which is why we restricted the sensitivity of candidate scenarios (with an optional exception to include UKESM1-0-LL).

## Data Records

The ClimateAF software version 1.1 as well as corresponding gridded databases at 30 arcsecond resolution (approx. 1 km) and 2.5 arcminute resolution (approx. 4 km) were deposited as an open access dataset on the Figshare repository^29^ (10.6084/m9.figshare.27905547). In addition, a constantly updated version of the database is available under the URL shortcut http://tinyurl.com/ClimateAF, where new historical climate grids from recent weather station data and new releases of future projections from AOGCMs will be posted.

## Technical Validation

**Fig. 4:**
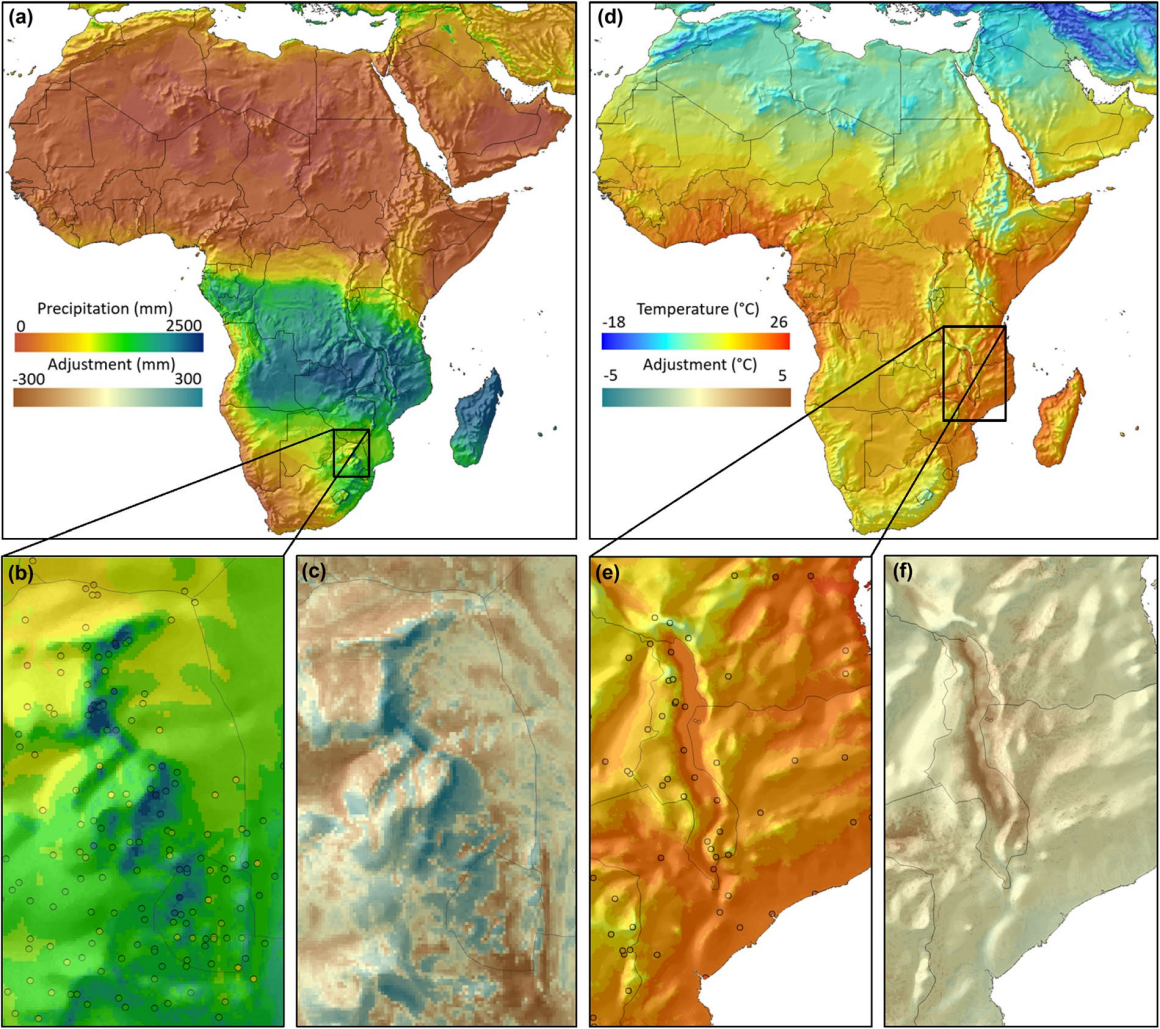
Example climate grid for January precipitation (**a**–**c**) and January mean minimum temperature (**d**–**f**), including a difference calculations (**c,f**) that highlight the effects of neural network fine-tuning. The color of circles in the inset indicates the weather station values (or residuals on the difference layer) on the same scale as the gridded data.

Visual inspection of the effect of neural network fine-tuning reveals that the methodological approach was capable of capturing local climate patterns that are not accounted for by standard interpolation methods. For example, the neural network was able to pick up rain shadows on the leeward side, and precipitation due to orographic lift on the windward side of mountains (Fig. 4a–c). During the southern hemisphere summer, easterly monsoon winds bring moist air from the Indian Ocean, and the inset shows that elevated terrain on the windward side of the mountain range experiences orographic rainfall, while leeward facing slopes show corresponding rain shadows, very closely tracking weather station values (Fig. 4b). The effect of the neural network fine tuning, relative to the initial thin plate spline interpolation (Fig. 4c) reveals that the initial grid generated with standard thin-plate spline interpolation gets corrected to higher precipitation values on the upper slopes of the mountain range, while that rain shadows are more evenly introduced on the leeward regions. This conforms to general expectations of weather patterns when oceanic air is lifted across mountain ranges.

Modifications of temperature grids by the neural network are generally minor, except for areas surrounding large lakes, indicating that thin-plate spline interpolation with an altitude covariate already provides excellent estimates for lapse-rate driven temperature gradients. In the example for January minimum temperature (Fig. 4d-f) a moderate ~2 °C adjustment is visible, putatively representing a lake effect on minimum temperature for Lake Malawi. The lake effect appears asymmetric, slightly shifted towards the east, arguably caused by prevailing monsoon winds from the east during this time of year.

The accuracy of monthly climate estimates for the 1961–1990 normal period, based on the independent “checkerboard” validation method that controls for spatial autocorrelations in weather station data was evaluated with three metrics (Table 3). The mean absolute error values (MAE) in original units of temperature are approximately 0.9 °C on average for the continent, and around 13 mm for precipitation. The proportion of variance explained in weather station data (R-squared), allows comparison of variables in different units, showing that it is more difficult to predict precipiation than temperature values accurately (Table 3). The Root Mean Square Error (RMSE) is a metric sensitive to large residuals and outliers (since they are squared as opposed to the MAE calculation), and reveals values slightly higher than the MAE statistic.

**Table 3:** Checkerboard cross-validation statistics for the neural network model.

Month	MAE			R^2^			RMSE		
	Tmin (°C)	Tmax (°C)	Prec (mm, %)	Tmin	Tmax	Prec	Tmin (°C)	Tmax (°C)	Prec (mm, %)
Jan	0.93	0.85	14, 20	0.98	0.98	0.91	1.28	1.21	24, 36
Feb	0.89	0.82	13, 19	0.98	0.98	0.90	1.20	1.13	22, 34
Mar	0.84	0.81	14, 21	0.97	0.98	0.85	1.19	1.16	26, 39
Apr	0.84	0.84	14, 23	0.97	0.97	0.82	1.17	1.19	29, 46
May	0.92	0.93	12, 25	0.97	0.96	0.85	1.27	1.29	26, 54
Jun	0.99	0.98	12, 26	0.97	0.96	0.90	1.34	1.33	26, 56
Jul	0.96	0.98	13, 22	0.97	0.96	0.91	1.33	1.37	31, 52
Aug	0.98	0.97	16, 22	0.96	0.96	0.90	1.38	1.36	36, 51
Sep	0.92	0.89	13, 21	0.95	0.95	0.93	1.31	1.24	25, 42
Oct	0.92	0.84	13, 24	0.95	0.95	0.87	1.31	1.22	25, 46
Nov	0.91	0.79	14, 24	0.96	0.97	0.82	1.26	1.16	27, 46
Dec	0.95	0.83	13, 21	0.97	0.98	0.90	1.28	1.16	23, 37
Average	0.92	0.88	13, 22	0.97	0.97	0.88	1.28	1.24	27, 45

Abbreviations refer to MAE, absolute mean error; R^2^ is the coefficient of determination and RMSE is the root mean squared error. Variable abbreviations are Tmin, mean monthly minimum temperature; Tmax, mean monthly maximum temperature; Prec, total monthly precipitation.

Once the baseline climate grids are incorporated into the *ClimateAF* software package, additional improvements of climate estimates are generated by downscaling of the 2.5 arcminute 1961–1990 baseline grids to any elevation of interest, using empirical lapse-rate based elevation adjustment for each variable, elevation and location. It should be noted that this additional evaluation was not an independent test because for the final gridded database included in the *ClimateAF* software package all stations were used in model development for the interpolated surfaces. For temperature variables, the lapse-rate based software adjustment to obtain a climate estimate of the exact elevation of the weather station, as opposed to using the value of grid cell in which the weather station is located (possibly with limited location precision) considerably improves temperature estimates (Table 4). For all regions of Africa, mean absolute errors of temperature estimate are around 0.5 °C based on scale-free point estimates by the ClimateAF software. Precipitation errors are more variable among regions, where the MAE expressed in percent is driven by the total precipitation a region receives. However, the MAE expressed as percentage of the total still reveals strong regional variation in statistical precision. Where monthly precipitation is very low, small deviations of the estimate result in high errors expressed as percent (e.g., ARP, Arabian Peninsula; SAH, Sahara). Another area with high error is the portion of the West Central Asia (WCA) IPCC region that is included in the study area and where station coverage is poor.

**Table 4:** Mean absolute error between observed and interpolated surfaces for monthly, seasonal or annual variables.

Region	Monthly				Seasonal		Annual	
	Tmin (°C)	Tmax (°C)	Tave (°C)	Prec (mm, %)	Tave (°C)	Prec (mm, %)	Tave (°C)	Prec (mm, %)
ARP	0.54	0.54	0.48	3, 38	0.45	8, 35	0.39	23, 24
CAF	0.36	0.35	0.30	9, 9	0.29	22, 8	0.27	69, 6
ESAF	0.44	0.44	0.34	8, 12	0.33	20, 10	0.30	69, 9
MDG	0.45	0.39	0.35	21, 17	0.34	49, 13	0.32	173, 12
MED	0.63	0.58	0.52	7, 19	0.5	19, 17	0.46	66, 15
NEAF	0.56	0.53	0.45	12, 16	0.43	31, 14	0.40	102, 12
SAH	0.59	0.48	0.48	3, 18	0.46	7, 14	0.41	23, 11
SEAF	0.45	0.49	0.41	16, 17	0.4	41, 14	0.38	139, 12
WAF	0.39	0.35	0.28	8, 9	0.27	20, 8	0.23	63, 6
WCA	0.59	0.56	0.51	8, 27	0.49	21, 23	0.45	76, 21
WSAF	0.51	0.58	0.49	4, 12	0.47	11, 11	0.44	36, 9
Africa	0.51	0.48	0.42	8, 13	0.4	20, 11	0.37	68, 9

Variable abbreviations include Tmin, Tmax, Tave: mean minimum, maximum and average temperature; Prec, total precipitation. Abbreviations for IPCC climate regions^19^ are ARP, Arabian-Peninsula; CAF, Central-Africa; ESAF, East-Southern Africa; MDG, Madagascar MED, Mediterranean; NEAF, North-Eastern-Africa; SAH, Sahara; SEAF, South-Eastern-Africa; WAF, Western-Africa; WCA, West Central Asia and; WSAF, West-Southern-Africa.

Comparing different time periods for climate variables, including monthly, seasonal, and annual estimates, it can be seen that the precision for longer seasonal and annual periods increases (Table 4). In general, the precision of climate estimates increases with the length of time that the variables represent, i.e., mean annual temperature estimates are more precise than seasonal variables, which are in turn are more precise than monthly variables. Although not evaluated specifically in this study, the same trend extends to long term averages, i.e., a 30-year normal estimate is more precise than a decadal or annual average for any variable (monthly, seasonal, or annual) e.g., see^39^.

**Fig. 5:**
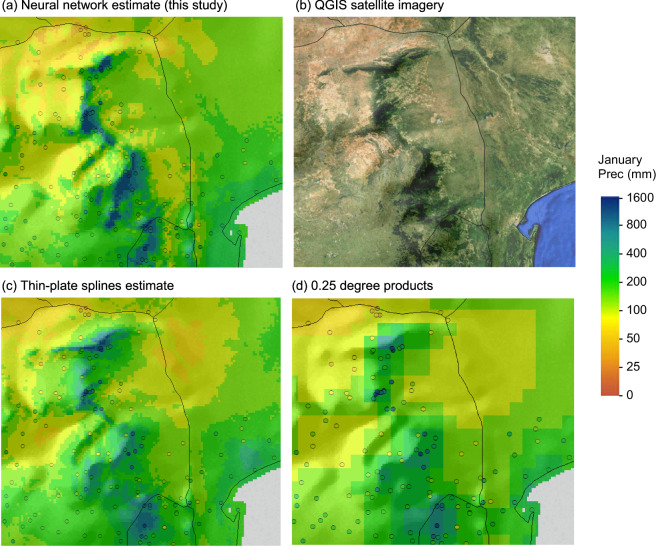
Comparison of interpolated climate grids for January precipitation, fine-tuned with deep neural networks from this study (**a**), with a QGIS Plugin for Copernicus Sentinel-2 imagery (**b**), the widely used thin-plate spline interpolation method (**c**), and a commonly used grid size for time series data (**d**). Weather station data is represented by open circles with fill values on the same scale as the climate interpolations.

The *ClimateAF* software package we provide is based on an equivalent methodology that was previously developed for North America^13^ and Europe^14^. Compared to those datasets, the MAE and R² metrics for the African dataset are broadly similar, indicating that the interpolation methods used here are approximately comparable in quality to the PRISM methodology. Like PRISM methodology, deep neural networks can capture local climate patterns that may not be optimally represented by standard interpolation techniques. For example, patterns of dense vegetation on windward (south east) facing slopes very closely track modeled precipitation patterns (e.g., Fig. 5a versus 5b), which standard interpolation techniques based on latitude, longitude and elevation alone cannot model (Fig. 5c). Similarly, lower resolution data products, such as CRU used to derive anomaly layers for this database, cannot capture fine-scale precipitation patterns (Fig. 5d), but are nevertheless valuable to generate historical time series with a change-factor calculation on the high-resolution baseline.

## Usage Notes

Users of our climate databases should keep in mind that climatic features such as rain shadows or lake and coastal influences are modeled at a scale of 2.5 arcminutes (approximately 4 km), suitable to broadly represent macroclimatic patterns in the landscape. Lapse-rate driven differences in temperature related variables along elevation gradients are represented at a finer scale, informative at a resolution of hundreds of meters in steep mountainous terrain. We should note, however, that all interpolated climate surfaces of this dataset are ultimately based on standard climate stations and consequently, microclimates that are driven by vegetation, water bodies, or topography at a scale of tens of meters are not represented.

For future projections, the 13 AOGCMs selected by Mahony, *et al*.^16^ globally conserve the climate uncertainty and sensitivity (magnitude of change) of the larger group of CMIP6 models. If possible, we recommend that all 13 models are used for assessment of uncertainty. However, this may not always be possible if assessments require significant computational resources. A common approach is to select a median, a pessimistic and an optimistic projection, i.e., a subset size of 3. By necessity, small subsets may not conserve uncertainty and sensitivity of the larger group of CMIP6 models as well. However, Table 5 provides a recommendation with minimal compromise for a user-determined number of models to assess uncertainty for different regions of Africa, as well as for the entire continent.

**Table 5:** Subsets of the projections with optimal representation variation in climate change projections for a given subset size according to, including additional selection criteria from Mahony, *et al*.^16^.

Subset size	IPCC reference region											
	ARP	CAF	ESAF	MDG	MED	NEAF	SAH	SEAF	WAF	WCA	WSAF	Africa
1	GISS	MRI	GFDL	MRI	CNRM	GISS	CNRM	GFDL	GISS	MRI	GFDL	GISS
2	EC	MPI	CNRM	ACC	EC	MIR	MPI	ACC	MPI	EC	CNRM	MPI
3	MRI	ACC	MPI	MIR	MPI	ACC	EC	MIR	EC	MPI	MIR	ACC
4	MPI	MIR	MIR	CNRM	GISS	GFDL	MRI	MPI	ACC	MIR	GISS	MIR
5	ACC	EC	GISS	GISS	MRI	MRI	ACC	EC	MIR	ACC	EC	MRI
6	CNRM	GFDL	EC	MPI	ACC	EC	GISS	MRI	CNRM	GFDL	MPI	EC
7	GFDL	CNRM	ACC	EC	MIR	MPI	MIR	CNRM	GFDL	CNRM	MRI	CNRM
8	MIR	GISS	MRI	GFDL	GFDL	CNRM	GFDL	GISS	MRI	GISS	ACC	GFDL

The IPCC reference regions^19^ for Africa as shown in Figure S1 include: ARP, Arabian-Peninsula; CAF, Central-Africa; ESAF, East-Southern Africa; MDG, Madagascar MED, Mediterranean; NEAF, North-Eastern-Africa; SAH, Sahara; SEAF, South-Eastern-Africa; WAF, Western-Africa; WCA, West Central Asia and; WSAF, West-Southern-Africa. For an equivalent table that includes the sensitive UKESM1-0-LL scenario, refer to Table 6.

For example, to assess uncertainty in West Africa (WAF) with a 3-model ensemble, users may choose: GISS (median), MPI (optimistic), and EC (pessimistic) from Table 5. Adding models (rows 4 to 8) will provide increasingly better representation of uncertainty in future predictions in multivariate space. That said, users may choose to add a high sensitivity model UKES to their model selection at their discretion (Table 6). If UKES is included, this scenario will almost always be chosen second, as the most “pessimistic” model of global warming. UKES is not a very likely outcome, but if users work with a larger ensemble set, a more sensitive model may be included as one possible outcome.

**Table 6:** Recommended subsets of projections as in Table 5, but including the sensitive UKESM1-0-LL scenario.

Subset size	IPCC reference region											
	ARP	CAF	ESAF	MDG	MED	NEAF	SAH	SEAF	WAF	WCA	WSAF	Africa
1	CNRM	GISS	EC	CNRM	CNRM	GISS	CNRM	CNRM	CNRM	CNRM	EC	CNRM
2	UKES	UKES	UKES	UKES	UKES	UKES	UKES	UKES	MPI	UKES	UKES	UKES
3	EC	MPI	MPI	MPI	EC	MIR	MPI	MPI	UKES	EC	MIR	MPI
4	MPI	EC	MIR	MIR	MPI	ACC	EC	MIR	EC	MPI	CNRM	GFDL
5	MRI	MIR	CNRM	GISS	GISS	GFDL	MRI	EC	GFDL	GFDL	GISS	ACC
6	ACC	ACC	GISS	ACC	MRI	MRI	ACC	GISS	MIR	MIR	MRI	EC
7	GISS	GFDL	MRI	MRI	ACC	EC	GISS	MRI	GISS	ACC	GFDL	MIR
8	MIR	CNRM	ACC	EC	MIR	MPI	MIR	GFDL	ACC	MRI	MPI	GISS
9	GFDL	MRI	GFDL	GFDL	GFDL	CNRM	GFDL	ACC	MRI	GISS	ACC	MRI

For larger ensembles with 7 or more AOGCMs, these sets in this table are recommended.

## Data Availability

The code for thin plate spline interpolation and fine tuning of climate grids with deep neural networks is publicly available under a Creative Commons Attribution 4.0 International license (CC BY 4.0), and are included in the Figshare repository^29^. While the *ClimateAF* software version corresponding to this paper has been deposited in an open access repository for unrestricted public access, the codebase for the graphical user interface and GIS functionality of the *ClimateAF* software is not publicly available. Besides the data available in the repository corresponding to this publication, the latest version of this software and gidded database can be anonymously downloaded at http://tinyurl.com/ClimateAF.
